# High Nuclear Expression of Yes-Associated Protein 1 Correlates With Metastasis in Patients With Breast Cancer

**DOI:** 10.3389/fonc.2021.609743

**Published:** 2021-02-25

**Authors:** Yoon Jin Cha, Soong June Bae, Dooreh Kim, Sung Gwe Ahn, Joon Jeong, Ja Seung Koo, Tae-Kyung Yoo, Woo-Chan Park, Ahwon Lee, Chang Ik Yoon

**Affiliations:** ^1^ Department of Pathology, Gangnam Severance Hospital, College of Medicine, Yonsei University, Seoul, South Korea; ^2^ Department of Surgery, Gangnam Severance Hospital, College of Medicine, Yonsei University, Seoul, South Korea; ^3^ Department of Pathology, Severance Hospital, College of Medicine, Yonsei University, Seoul, South Korea; ^4^ Division of Breast Surgery, Department of Surgery, College of Medicine, Seoul St Mary’s Hospital, The Catholic University of Seoul, Seoul, South Korea; ^5^ Department of Pathology, Seoul St Mary’s Hospital, College of Medicine, The Catholic University of Seoul, Seoul, South Korea

**Keywords:** Yes-associated protein 1, breast cancer, prognosis, metastasis, triple-negative breast cancer

## Abstract

**Background:**

Yes-associated protein 1 (YAP1) is a transcription factor regulated by the Hippo pathway and functions as an oncogene in various solid tumors under dysregulated Hippo pathway. However, the role of YAP1 in breast cancer remains controversial. Here, we investigated the impact of different levels of nuclear YAP1 expression on the clinical characteristics and survival outcome in patients with breast cancer.

**Patients and Methods:**

Retrospectively obtained 455 breast tumor samples at Gangnam Severance Hospital were examined for YAP1 expression by immunohistochemistry, and the clinical data were analyzed. External validation was performed using a retrospective cohort and tissues in 482 patients from Severance Hospital.

**Results:**

High nuclear YAP1 expression was associated with hormone receptor negativity and aggressive tumor behavior, including lymph node metastasis, high Ki67 labeling index and inferior distant metastasis-free survival (DMFS, hazard ratio [HR] 2.271, 95% confidence intervals [CIs] 1.109–4.650, *P* = 0.0249), and also confirmed inferior disease free survival (HR 3.208, 95% CIs 1.313–7.833, *P* = 0.0105) in external validation cohort. In patients with triple-negative breast cancer (TNBC), high nuclear YAP1 expression was an independent significant determinant of poor DMFS (HR 2.384, 95% CIs 1.055–5.386, *P* = 0.0367).

**Conclusion:**

Our findings suggest that nuclear YAP1 expression is a biomarker of adverse prognosis and a potential therapeutic target in patients with breast cancer, especially in TNBC.

## Background

Breast cancer is the most common cancer among women worldwide, and approximately 15 million people are diagnosed with this disease each year. In recent years, owing to advanced treatment modalities and the identification of new drug targets, breast cancer mortality has decreased by approximately 2.3% per year (1). So far, well known biomarkers such as including ER and HER2, as well as others, including pSTAT3 expression, LDH, and tumor-infiltrating lymphocytes, etc. – have been studied to elucidate breast cancer biology and survival outcome (2–4), further endeavor is still required to understand tumor nature. Despite these improvements, metastatic breast cancer still results in poor survival. In order to further enhance treatment efficacy, new drug targets with specific roles in metastatic cascades must be unveiled.

Lymph node metastasis is the most important prognostic factor in breast cancer, and is associated with high relapse and mortality (5–8). The mechanisms underlying lymph node metastasis are still poorly understood. It has been reported that the transcriptional co-activator, Yes-associated protein 1 (YAP1), plays an important role in lymph node metastasis (9). Enhanced YAP1 activity increases fatty acid oxidation, ultimately leading to lymph node metastasis. The Hippo signaling regulates organ size and development (10), and restricts transcription co-factor YAP1 and transcriptional coactivator with PDZ-binding motif (TAZ) by cytoplasmic retention followed by protein degradation (11). However, dysregulated Hippo signaling results in the nuclear accumulation of non-phosphorylated YAP1 and TAZ. These interact with transcriptional enhanced associated domain (TEAD)-containing transcription factors in nucleus, promoting the expression of genes related to cell proliferation and epithelial-mesenchymal transition (EMT) (12, 13). Moreover, YAP1 was reported to induce TEAD-dependent focal adhesion kinase phosphorylation, ultimately promoting tumor invasiveness (14). Activated YAP1 contributes to cancer development by promoting a malignant tumor phenotype. In particular, YAP1 stimulates cancer stem cell proliferation and epithelial-mesenchymal transition, induces drug resistance, inhibits apoptosis, and promotes tumor overgrowth (10, 15–18). Several studies have reported a correlation of YAP1 expression with aggressive clinical characteristics and low survival (19–29). This evidence suggests that YAP1 is a potential therapeutic target, and that its pharmacologic or genetic inactivation may suppress tumor progression and improve drug sensitivity. However, the association between clinical data and YAP1 expression in patients with breast cancer has been poorly explored. In addition, the possible clinical relevance of YAP1 subcellular localization is not clearly defined.

The aim of this study was to correlation between the level of nuclear YAP1 expression and the clinical characteristics and survival rates of patients with breast cancer. The impact of YAP1 expression on survival was also evaluated in patients with triple negative breast cancer (TNBC).

## Materials and Methods

### Patients

We retrospectively collected the tumor tissues from the patients undergoing primary curative surgery for breast cancer at the Gangnam Severance Hospital in Seoul, Korea from February 1992 to April 2017 and at the Severance Hospital in Seoul, Korea from January 2000 to December 2010. A validation cohort consisted of 482 patients from the Severance Hospital. Inclusion and exclusion criteria were as follows:

1) Inclusion criteria:
  - Patients age ≥ 20 years
  - Breast cancer confirmed by pathologic diagnosis (stage I–III)
  - Available YAP1 immunohistochemical staining with tissue microarray
2) Exclusion criteria:
  - Any other carcinoma *in situ*
  - Other cancer history (except thyroid cancer and carcinoma in situ)
  - Not assessable electronic medical record

All subjects were diagnosed with stage I–III primary breast cancer. All patient treatments were performed according to standard protocols. The following data were collected: age at surgery, tumor size, lymph node status, histological grade (HG), status of estrogen receptor (ER), status of progesterone receptor (PR), status of human epidermal growth factor receptor-2 (HER2), lymphovascular invasion (LVI), Ki67 leveling index, tumor-infiltrating lymphocytes (TILs), treatment modalities, recurrence, and death. Tumor HG was determined by applying the modified Scarff-Bloom-Richardson grading system. The study protocol was approved by the institutional review board (IRB) of the Gangnam Severance Hospital (local IRB No. 3-2019-0188). The need for informed consent was waived under the approval of the IRB due to the retrospective design.

## Immunohistochemistry (IHC) and Molecular Subtyping

As previously described (30), 3-µm thick tissue sections were cut from formalin-fixed paraffin-embedded (FFPE) tissue microarray (TMA) blocks. After deparaffinization and rehydration with xylene and alcohol graded solutions, respectively, IHC was performed by using a Ventana Discovery XT Automated Slide Stainer (Ventana Medical System, Tucson, AZ, USA). Cell Conditioning 1 (CC1) buffer (citrate buffer, pH 6.0; Ventana Medical System) was used for antigen retrieval. The appropriate positive and negative controls were included.

IHC staining was evaluated with light microscopy (BX53 upright microscope, Olympus, Tokyo, Japan). Nuclear staining values of 1% or higher were considered indicative of ER (clone SP1; dilution 1:100; Thermo Scientific, San Diego, CA, USA) and PR (clone PgR; dilution 1:50; DAKO, Glostrup, Denmark) positivity (31). HER2 (polyclonal; dilution 1:1500; DAKO) staining was interpreted based on the 2018 American Society of Clinical Oncology/College of American Pathologists guidelines (32). Only samples with strong and circumferential membranous HER2 immunoreactivity (3+) were considered positive, while those with 0 and 1+ HER2 staining were considered negative. Cases with equivocal HER2 expression (2+) were further evaluated for HER-2 gene amplification by silver *in situ* hybridization (SISH). Positive nuclear Ki-67 (clone MIB; dilution 1:1,000; Abcam, Cambridge, UK) staining was assessed based on the percentage of positive tumor cells, defined as Ki-67 labeling index.

The specimens were categorized as follows: i) Luminal/HER2-negative (ER- and/or PR-positive and HER2-negative); ii) HER2-positive (HER2-positive regardless of ER and PR status); iii) TNBC (ER-, PR-, and HER2-negative).

## Evaluation of Nuclear YAP1 Expression by Tissue Microarray and IHC Staining

Hematoxylin and eosin-stained slides from the resected breast cancer specimens were examined, and the representative areas were marked. The matched tissue cores (2 mm) were extracted from FFPE tumor blocks and placed into 5 × 10 recipient TMA blocks.

**Figure 1:**
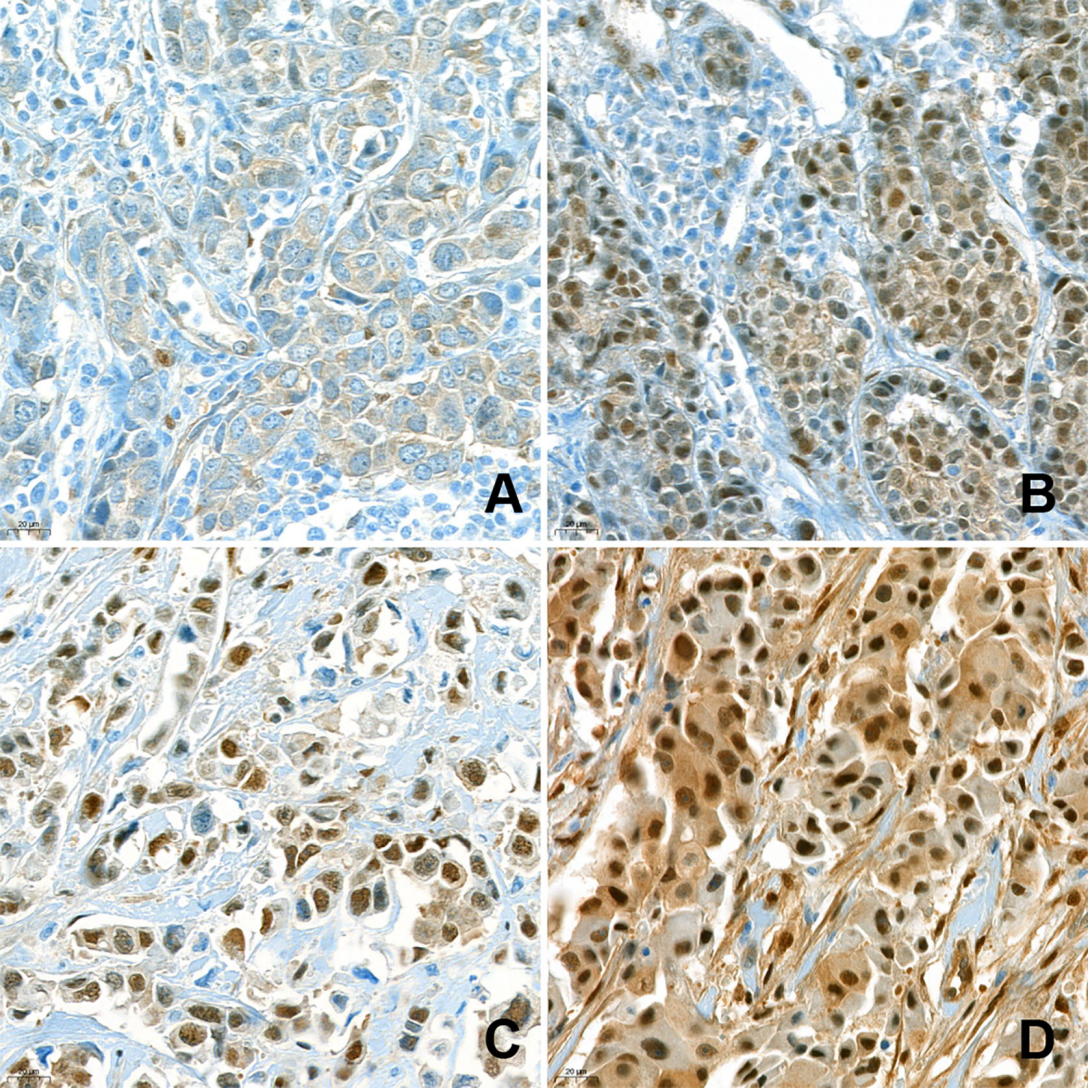
Immunohistochemical analysis of nuclear YAP1 expression. Nuclear YAP1 expression was evaluated in high-power fields (400× magnification) by an experienced pathologist. The samples were classified as negative **(A)**, 1+ **(B)**, 2+ **(C)**, and 3+ **(D)**, based on the intensity of YAP1 nuclear staining.

For IHC, each TMA slide was stained with anti-YAP1 antibody (clone 63.7; dilution 1:200; Santa Cruz Biotechnology, Dallas, TX, USA). After staining, nuclear YAP1 expression was assessed by a pathologist (400× magnification). YAP1 expression was evaluated in both the cytoplasm and nuclei of the tumor cells. Cytoplasmic staining was evaluated by the H-score, which was obtained by multiplying staining intensity (0, 1, 2, or 3) by percentage of stained area (%). As nuclear expression was rare and mostly focal, only the intensity of nuclear staining was examined (0, 1+, 2+, 3+), regardless of the corresponding cytoplasmic staining. The intensity of nuclear staining of the myoepithelial cells was assigned a value of 2+ and used as an internal control. Weaker and stronger signals were assigned a value of 1+ and 3+, respectively. Negative and weak (1+) nuclear staining were considered indicative of low expression, while moderate (2+) and strong (3+) nuclear expression were indicative of high expression (**Figure 1**). The IHC results were interpreted blindly, without any information regarding clinical parameters or outcomes.

## Statistical Analysis

Distant metastasis-free survival (DMFS) was defined as the time from the primary curative surgery to the first breast cancer-derived distant metastasis, or death due to any cause, or end of follow-up. Overall survival (OS) was defined as the time from the primary curative surgery to the end of follow-up, or death due to any cause. Disease-free survival (DFS) was defined as the time from the primary curative surgery to cancer recurrence, second cancer, or death. The data of patients who did not exhibit relevant events were censored at the end of follow-up.

The continuous variables between the two groups were compared using the Student’s *t*-test or the Mann-Whitney test. The categorical variables were compared by using the *Chi*-square test or the Fisher’s exact test. Survival curves were obtained by the Kaplan-Meier method and two-group comparisons were made using log-rank test. Univariate and multivariate Cox proportional hazard models were used to identify the factors associated with survival outcome (DMFS and OS). The variables showing statistically significant differences in the univariate analysis were used in the multivariate Cox proportional hazard models.

Statistical analysis was performed by using SPSS version 24 (SPSS: Chicago, IL, USA) software. The threshold for statistical significance was set at *P <*0.05, with a 95% confidence interval not including 1.

## Results

### Impact of Nuclear YAP1 Expression on the Baseline Characteristics of Patients With Breast Cancer

A total of 455 breast cancer patients at Gangnam Severance Hospital were included in this study. The median age was 50 (25–86) years. The median DFS and OS were 59 (10–325) and 60 (12–325) months. Low and high nuclear YAP1 expression were found in the tumors of 344 (75.6%) and 111 (24.4%) patients, respectively. The clinical characteristics were examined in relation to nuclear YAP1 expression (**Table 1**). High nuclear YAP1 expression was associated with aggressive tumor features, including hormone receptor negativity, high HG, lymph node metastasis, and high Ki67 expression. Patients were classified into three subtypes based on IHC analysis: luminal/HER2-negative (243 patients), HER2-positive (62 patients), and TNBC (146 patients). High nuclear YAP1 expression was associated with the TNBC subtype.

**Table 1:** Clinical characteristics in relation to nuclear YAP1 expression.

	YAP1-low, n = 344 (%)	YAP1-high, n = 111 (%)	*P* value
**Age (year, mean ± SD)**	50.78 ± 10.47	49.51 ± 9.50	0.256
**HR**			<0.001
**Positive**	238 (69.2)	35 (31.5)	
**Negative**	106 (30.8)	76 (68.5)	
**HER2** **^a^**			0.301
**Positive**	50 (14.5)	12 (10.8)	
**Negative**	290 (84.3)	99 (89.2)	
**Missing**	4 (1.2)	0	
**HG** **^a^**			0.005
**I, II**	223 (64.8)	54 (48.6)	
**III**	115 (33.4)	52 (46.8)	
**Missing**	6 (1.7)	5 (4.5)	
**Subtype** **^a^**			<0.001
**Luminal/HER2(**−**)**	210 (61.0)	33 (29.7)	
**HER2 (+)**	50 (14.5)	12 (10.8)	
**TNBC**	80 (23.3)	66 (59.9)	
**Missing**	4 (1.2)	0	
**Tumor size** **^a^**			0.298
**≤2 cm**	174 (50.6)	50 (45.0)	
**>2 cm**	169 (49.1)	61 (55.0)	
**Missing**	1 (0.3)	0	
**Lymph node metastasis** **^a^**			0.602
**Negative**	231 (67.2)	72 (64.9)	
**Positive**	111 (32.3)	39 (35.1)	
**Missing**	2 (0.6)	0	
**Ki67 (%)** **^a^**			0.001
**≤20%**	258 (75.0)	62 (55.9)	
**>20%**	74 (21.5)	40 (36.0)	
**Missing**	12 (3.5)	9 (8.1)	
**Lymphovascular invasion** **^a^**			0.413
**Negative**	268 (77.9)	81 (73.0)	
**Positive**	55 (16.0)	21 (18.9)	
**Missing**	21 (6.1)81	9 (8.1)	
**TILs (%, mean ± SD)** **^a^**	33.84 ± 26.15 (n = 164)	33.54 ± 30.77 (n = 72)	0.943

SD, standard deviation; HR, hormone receptor; HER-2, human epidermal growth factor receptor-2; HG, histological grade; TNBC, triple negative breast cancer; TILs, tumor-infiltrating lymphocytes.

a Percentages calculated without missing values.

Validation cohort included 482 patients at Severance Hospital. Median DFS and OS were 65 (5–139) and 65 (12–241) months. Of the 482 patients, 428 (88.8%) exhibited low nuclear YAP1 expression, and 54 (11.2%) exhibited high nuclear YAP1 expression. Clinical characteristics were compared to nuclear YAP1 expression in **Supplementary Table 1**. Also, high nuclear YAP1 expression was related to TNBC subtype.

### Prognostic Significance of Nuclear YAP1 Expression

**Figure 2:**
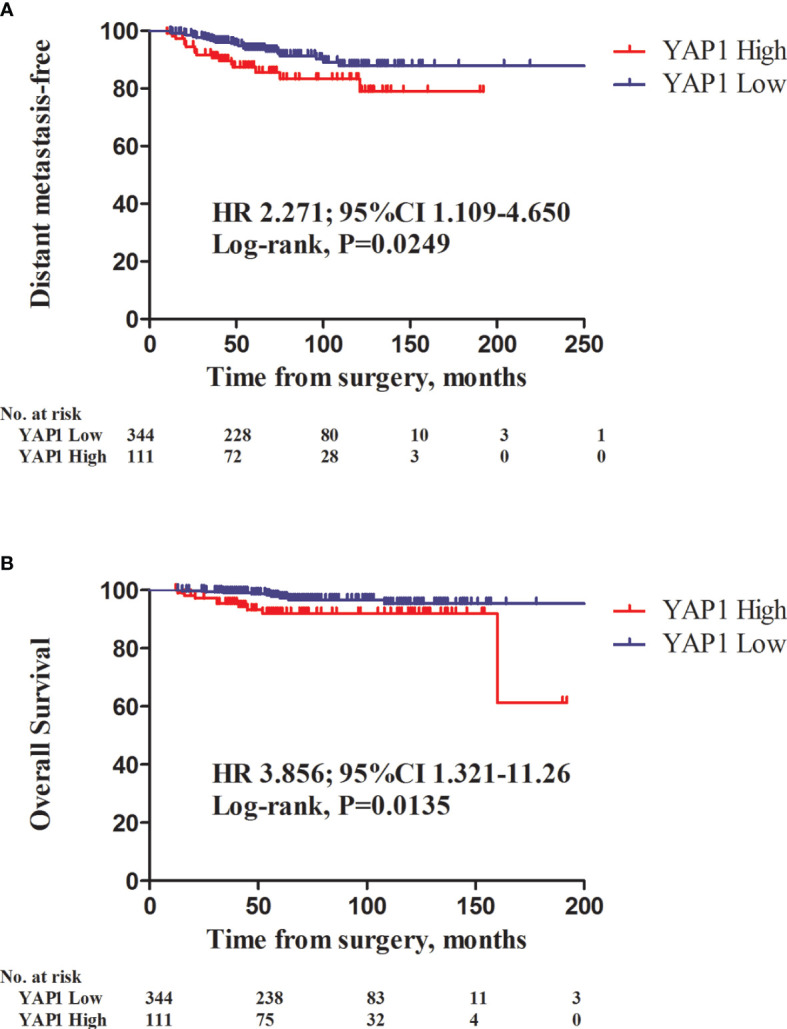
Kaplan-Meier survival curves of distant metastasis-free survival (DMFS) and overall survival (OS) in relation to nuclear YAP1 expression. Patients with high nuclear YAP1 expression exhibited poor DMFS and overall survival (OS) (**A**, HR 2.271, 95% CI 1.109–4.650, *P* = 0.0249; **B**, HR 3.856, 95%CI 1.321–11.26; *P* = 0.0135, log-rank test, respectively).

There were 41 patients with developing distant metastasis. Among them, 14 had bone metastasis, 11 lung metastasis, five liver metastasis, three brain metastasis, while 17 had developed metastases to other sites (including duplication). There were 18 mortality events. High nuclear YAP1 expression was significantly associated with decreased distant metastasis-free survival (DMSF) [**Figure 2A**; hazard ratio (HR), 2.271, 95% confidence intervals (CIs) 1.109–4.650, *P* = 0.0249, log rank test], and was a significant predictor of poor overall survival (**Figure 2B**; HR 3.856, 95% CIs 1.321–11.26, *P* = 0.0135, log rank test).

Negative hormone receptor status, tumor size >2 cm, and high nuclear YAP1 expression were significantly associated with decreased DMFS, as assessed by univariate analysis (**Table 2**). High nuclear YAP1 expression was still a significant determinant of decreased DMFS after adjustment for hormone receptor status, tumor size, and nuclear YAP1 expression by the Cox proportional hazards model (HR 1.893, 95% CIs 1.009–3.552, *P* = 0.047).

**Table 2:** Hazard ratios (HRs) and 95% confidence intervals (CIs) for distant metastasis-free survival (DMFS).

	Univariate analysis		Multivariate analysis	
	HRs (95% CIs)	*P* value	HRs (95% CIs)	*P* value
**Age**	0.983 (0.953–1.014)	0.284		
**HR**		0.015		0.158
**Negative**	Reference		Reference	
**Positive**	0.449 (0.236–0.854)		0.607 (0.303–1.215)	
**HER2**		0.295		
**Negative**	Reference			
**Positive**	0.575 (0.204–1.619)			
**HG**		0.101		
**I, II**	Reference			
**III**	1.670 (0.904–3.083)			
**Tumor size**		0.009		0.012
**≤2 cm**	Reference		Reference	
**>2 cm**	2.525 (1.263–5.049)		2.426 (1.211–4.861)	
**Lymph node metastasis**		0.051		
**Negative**	Reference			
**Positive**	1.840 (0.996–3.397)			
**YAP1 expression**		0.028		0.047
**Low**	Reference		Reference	
**High**	2.020 (1.078–3.784)		1.893 (1.009–3.552)	
**Ki67 (%)**		0.237		
**≤20%**	Reference			
**>20%**	1.530 (0.756–3.095)			
**Lymphovascular invasion**		0.391		
**Negative**	Reference			
**Positive**	1.411 (0.643–3.099)			
**TILs**	0.988 (0.971–1.006)	0.200		

HR, hormone receptor; HER-2, human epidermal growth factor receptor-2; HG, histologic grade; TILs, tumor-infiltrating lymphocytes.

In the univariate Cox proportional hazard model, negative hormone receptor status, high histologic grade, tumor size > 2 cm, and high nuclear YAP1 expression were found to be significant prognostic factors for OS (**Supplementary Table 2**). However, in the multivariate analysis, nuclear YAP1 expression was not a significant predictor of OS (**Supplementary Table 2**, HR 1.576, 95% CIs 0.616–4.034, *P* = 0.343).

In validation cohort, high nuclear YAP1 expression was significantly predictive of decreased DFS (**Supplementary Figure 1**; HR, 3.208, 95% CIs 1.313–7.833, *P* = 0.0105, log rank test). ER negativity, PR negativity, tumor size >2 cm, lymph node metastasis, and high nuclear YAP1 expression were significant factors in the multivariate analysis of DFS (**Supplementary Table 3**). When adjusted for other factors, high nuclear YAP1 expression was a significant factor in reduced DFS (HR 2.112, 95% CIs 1.083–4.119, *P* = 0.028).

### Prognostic Significance of Nuclear YAP1 Expression in TNBC Patients

**Figure 3:**
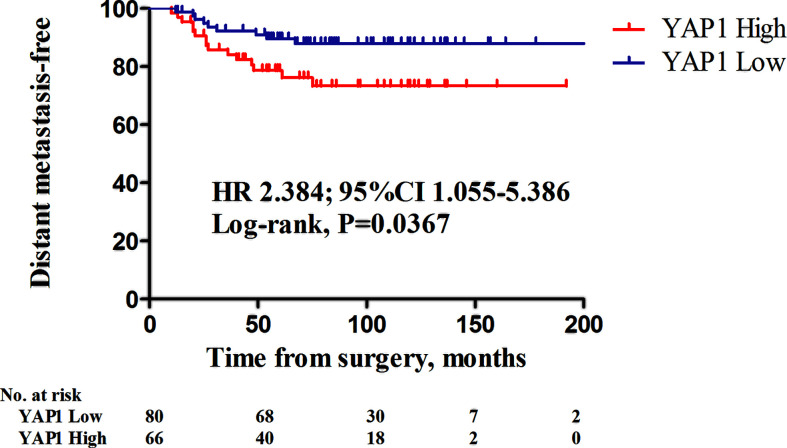
Kaplan-Meier survival curves of distant metastasis-free survival (DMFS) in relation to nuclear YAP1 expression in triple-negative breast cancers (TNBCs). Patients with high nuclear YAP1 expression exhibited poor DMFS (HR 2.384, 95% CI 1.055–5.386, *P* = 0.0367; log-rank test).

The impact of nuclear YAP1 expression on survival and clinical characteristics was evaluated in patients with TNBC (**Supplementary Table 4**). Of the 146 TNBC patients, 80 (54.8%) had tumors with low nuclear YAP1 expression, while 66 (45.2%) had tumors with high nuclear YAP1 expression. In TNBC patients, the clinical characteristics were not significantly affected by the level of nuclear YAP1 expression. However, high nuclear YAP1 expression was associated with poor DMFS (**Figure 3**; HR 2.384, 95% CIs 1.055–5.386, *P* = 0.0367, log rank test). Lymph node metastasis and high nuclear YAP1 expression were significant determinants of poor DMFS in the univariate analysis (**Supplementary Table 5**). Adjustment for significant factors in the univariate analysis confirmed that high nuclear YAP1 expression was significantly associated with DMFS (HR 2.329, 95% CIs 1.016–5.339, *P* = 0.046).

## Discussion

In this study, the nuclear expression of YAP1 was evaluated in a large number of breast cancer specimens, was found to be significantly associated with the occurrence of distant metastasis (HR 2.271, 95%CIs 1.109–4.650, *P* = 0.0249). Furthermore, nuclear YAP1 expression was a strong determinant of metastasis in TNBC (HR 2.384, 95%CIs 1.055–5.386, *P* = 0.0367), an aggressive subtype of breast cancer. These findings indicate that targeted therapy for YAP1 may potentially improve the survival outcomes, particularly metastasis, of the patients with breast cancer.

The activation of YAP1, along with that of another transcriptional co-activator TAZ, is associated with dysregulated Hippo signaling (33, 34). YAP1 overexpression promotes EMT, inhibits apoptosis, and induces growth factor-independent cell proliferation. Based on these findings, it was speculated that YAP1 may play a role as a proto-oncogene (23). In addition, several studies have reported a negative impact of YAP1 activation on the survival of patients with gastric, colorectal, ovarian, bladder, and non-small cell lung cancer (22, 29, 35–37).

Currently, the role of YAP1 in breast cancer remains controversial. For instance, Lehn and colleagues reported that YAP1 expression is inversely correlated with HG and tumor cell proliferation, and that low YAP1 mRNA levels are associated with decreased recurrence-free survival and tamoxifen-resistance in luminal A subtype breast cancer (38). Also, some have reported that YAP1 promotes cell proliferation, tumorigenesis, EMT, and drug resistance, and is associated with TP53 mutation, ER negativity, and poor survival (18, 39). However, several other studies have suggested that survival is not significantly affected by YAP1 expression in breast cancer (39, 40), or YAP1 may in fact act as a tumor suppressor (26, 41). Among the most of previous studies mentioned above, the specific argument on subcellular localization of YAP1 in previous studies were unclear. Although Vlug et al. showed upregulated YAP1 expression in invasive lobular carcinoma, but its prognostic impact was not evaluated (39). Kim et al. evaluated the differential expression of YAP and phosphorylated YAP in the each of molecular subtypes of breast cancer and found that nuclear YAP expression was associated with shorter survival (20). However, in previous study, nuclear YAP expression in multivariate analysis did not have statistical significance. Given that nuclear YAP1 expression is a surrogate marker of an activated form of YAP1 (9), we focused on the nuclear YAP1 expression in this study, and found that nuclear YAP1 expression is an independent prognostic predictor for distant metastasis, particularly in the TNBC patients.

TNBC is simply defined as a breast cancer that satisfied the ER, PR, and HER2 negativities, however it is not a uniform subtype rather a complex heterogeneous collection of molecularly different subtypes (42). Unlike hormone receptor-positive type or HER2-type breast cancer, there is currently limited therapeutic targets for TNBC, Although immune checkpoint inhibitors are now applied in TNBC in combination with chemotherapy (43, 44), demanding on targeted therapy is still existed, and YAP1 could be the one of candidate of potential therapeutic target.

Our study has several limitations. First, it was a retrospective study. Second, the TNBC subtype was overrepresented due to selection bias during sample collection. Third, TMA slide staining may underestimate the rate of YAP1 positivity compared to whole-slide examination. Although our study is difficult to compare with previous IHC studies (20, 26, 38, 40, 41, 45), as these did not clarify whether the employed antibodies were specific for phosphorylated YAP1, nor was the intracellular localization of the antigen established, we specifically examined the nuclear YAP1 expression and confirmed its prognostic effect. In our study, no correlations were found between cytosolic YAP1 staining and survival (data not shown). Despite these limitations, the results demonstrated that nuclear YAP1 expression was a clinical prognostic factor in breast cancer, especially TNBC.

In conclusion, we suggested that nuclear YAP1 expression is a clinical prognostic factor for breast cancer. In addition, YAP1 is a potentially valuable therapeutic target for patients with breast cancer, especially in TNBC.

## Data Availability Statement

The raw data supporting the conclusions of this article will be made available by the authors, without undue reservation.

## Ethics Statement

All procedures performed in studies involving human participants were in accordance with the ethical standards of the institutional and/or national research committee and with the 1964 Helsinki Declaration and its later amendments or comparable ethical standards. The protocol was approved by the institutional review board (Local IRB number: 3-2019-0188) of Gangnam Severance Hospital. The need for informed consent was waived under the approval of the IRB due to the retrospective design.

## Author Contributions

YC and CY had full access to all of data in the study and take responsibility for the integrity of the data and accuracy of the data analysis. Conceptualization, YC and CY. Data curation, YC, SB, DK, JK, T-KY, W-CP, AL, and CY. Funding acquisition, YC. Investigation, YC, SB, DK, SA, JJ, and CY. Methodology, YC and CY. Resources, JJ and JK. Formal analysis, YC and CY. Supervision, JJ. Writing—original draft, YC and CY. All authors contributed to the article and approved the submitted version.

## Funding

This study was supported by a faculty research grant from the Yonsei University College of Medicine (6-2018-0080).

## Conflict of Interest

The authors declare that the research was conducted in the absence of any commercial or financial relationships that could be construed as a potential conflict of interest.

## References

[1] Jemal A Siegel R Ward E Murray T Xu J Thun MJ. Cancer statistics, 2007. CA Cancer J Clin (2007) 57(1):43–66. 10.3322/canjclin.57.1.43 17237035

[2] Jurisic V Radenkovic S Konjevic G. The Actual Role of LDH as Tumor Marker, Biochemical and Clinical Aspects. Adv Exp Med Biol (2015) 867:115–24. 10.1007/978-94-017-7215-0_8 26530363

[3] Radenkovic S Konjevic G Gavrilovic D Stojanovic-Rundic S Plesinac-Karapandzic V Stevanovic P. pSTAT3 expression associated with survival and mammographic density of breast cancer patients. Pathol Res Pract (2019) 215(2):366–72. 10.1016/j.prp.2018.12.023 30598340

[4] Lotfinejad P Asghari Jafarabadi M Abdoli Shadbad M Kazemi T Pashazadeh F Sandoghchian Shotorbani S. Prognostic Role and Clinical Significance of Tumor-Infiltrating Lymphocyte (TIL) and Programmed Death Ligand 1 (PD-L1) Expression in Triple-Negative Breast Cancer (TNBC): A Systematic Review and Meta-Analysis Study. Diagnostics (Basel) (2020) 10(9):704. 10.3390/diagnostics10090704 PMC7554852 32957579

[5] Ferris RL Lotze MT Leong SP Hoon DS Morton DL. Lymphatics, lymph nodes and the immune system: barriers and gateways for cancer spread. Clin Exp Metastasis (2012) 29(7):729–36. 10.1007/s10585-012-9520-2 PMC3485421 22851005

[6] Carter CL Allen C Henson DE. Relation of tumor size, lymph node status, and survival in 24,740 breast cancer cases. Cancer (1989) 63(1):181–7. 10.1002/1097-0142(19890101)63:1<181::aid-cncr2820630129>3.0.co;2-h 2910416

[7] Atkinson EN Brown BW Montague ED. Tumor volume, nodal status, and metastasis in breast cancer in women. J Natl Cancer Inst (1986) 76(2):171–8. 10.1093/jnci/76.2.171 3456058

[8] https://seer.cancer.gov/archive/publications/survival/. SEER Survival Monograph: Cancer Survival Among Adults: US SEER Program, 1988-2001, Patient and Tumor Characteristics.

[9] Lee CK Jeong SH Jang C Bae H Kim YH Park I. Tumor metastasis to lymph nodes requires YAP-dependent metabolic adaptation. Science (2019) 363(6427):644–9. 10.1126/science.aav0173 30733421

[10] Pan D. The hippo signaling pathway in development and cancer. Dev Cell (2010) 19(4):491–505. 10.1016/j.devcel.2010.09.011 20951342 PMC3124840

[11] Meng Z Moroishi T Guan KL. Mechanisms of Hippo pathway regulation. Genes Dev (2016) 30(1):1–17. 10.1101/gad.274027.115 26728553 PMC4701972

[12] Zhao B Kim J Ye X Lai ZC Guan KL. Both TEAD-binding and WW domains are required for the growth stimulation and oncogenic transformation activity of yes-associated protein. Cancer Res (2009) 69(3):1089–98. 10.1158/0008-5472.Can-08-2997 19141641

[13] Zhao B Wei X Li W Udan RS Yang Q Kim J. Inactivation of YAP oncoprotein by the Hippo pathway is involved in cell contact inhibition and tissue growth control. Genes Dev (2007) 21(21):2747–61. 10.1101/gad.1602907 PMC2045129 17974916

[14] Shen J Cao B Wang Y Ma C Zeng Z Liu L. Hippo component YAP promotes focal adhesion and tumour aggressiveness via transcriptionally activating THBS1/FAK signalling in breast cancer. J Exp Clin Cancer Res (2018) 37(1):175. 10.1186/s13046-018-0850-z 30055645 PMC6064138

[15] Cheng H Zhang Z Rodriguez-Barrueco R Borczuk A Liu H Yu J. Functional genomics screen identifies YAP1 as a key determinant to enhance treatment sensitivity in lung cancer cells. Oncotarget (2016) 7(20):28976–88. 10.18632/oncotarget.6721 PMC5045371 26716514

[16] Johnson R Halder G. The two faces of Hippo: targeting the Hippo pathway for regenerative medicine and cancer treatment. Nat Rev Drug Discov (2014) 13(1):63–79. 10.1038/nrd4161 24336504 PMC4167640

[17] Mo JS Park HW Guan KL. The Hippo signaling pathway in stem cell biology and cancer. EMBO Rep (2014) 15(6):642–56. 10.15252/embr.201438638 PMC4197875 24825474

[18] Zhao B Lei QY Guan KL. The Hippo-YAP pathway: new connections between regulation of organ size and cancer. Curr Opin Cell Biol (2008) 20(6):638–46. 10.1016/j.ceb.2008.10.001 PMC3296452 18955139

[19] Kim DH Kim SH Lee OJ Huang SM Kwon JL Kim JM. Differential expression of Yes-associated protein and phosphorylated Yes-associated protein is correlated with expression of Ki-67 and phospho-ERK in colorectal adenocarcinoma. Histol Histopathol (2013) 28(11):1483–90. 10.14670/hh-28.1483

[20] Kim SK Jung WH Koo JS. Yes-associated protein (YAP) is differentially expressed in tumor and stroma according to the molecular subtype of breast cancer. Int J Clin Exp Pathol (2014) 7(6):3224–34. PMC4097213 25031743

[21] Lee KW Lee SS Kim SB Sohn BH Lee HS Jang HJ. Significant association of oncogene YAP1 with poor prognosis and cetuximab resistance in colorectal cancer patients. Clin Cancer Res (2015) 21(2):357–64. 10.1158/1078-0432.Ccr-14-1374 PMC4513664 25388162

[22] Liu JY Li YH Lin HX Liao YJ Mai SJ Liu ZW. Overexpression of YAP 1 contributes to progressive features and poor prognosis of human urothelial carcinoma of the bladder. BMC Cancer (2013) 13:349. 10.1186/1471-2407-13-349 23870412 PMC3750259

[23] Overholtzer M Zhang J Smolen GA Muir B Li W Sgroi DC. Transforming properties of YAP, a candidate oncogene on the chromosome 11q22 amplicon. Proc Natl Acad Sci U S A (2006) 103(33):12405–10. 10.1073/pnas.0605579103 PMC1533802 16894141

[24] Wang X Su L Ou Q. Yes-associated protein promotes tumour development in luminal epithelial derived breast cancer. Eur J Cancer (2012) 48(8):1227–34. 10.1016/j.ejca.2011.10.001 22056638

[25] Xu MZ Yao TJ Lee NP Ng IO Chan YT Zender L. Yes-associated protein is an independent prognostic marker in hepatocellular carcinoma. Cancer (2009) 115(19):4576–85. 10.1002/cncr.24495 PMC2811690 19551889

[26] Yuan M Tomlinson V Lara R Holliday D Chelala C Harada T. Yes-associated protein (YAP) functions as a tumor suppressor in breast. Cell Death Differ (2008) 15(11):1752–9. 10.1038/cdd.2008.108 18617895

[27] Han SX Bai E Jin GH He CC Guo XJ Wang LJ. Expression and clinical significance of YAP, TAZ, and AREG in hepatocellular carcinoma. J Immunol Res (2014) 2014:261365. 10.1155/2014/261365 24860833 PMC4016924

[28] Fitamant J Kottakis F Benhamouche S Tian HS Chuvin N Parachoniak CA. YAP Inhibition Restores Hepatocyte Differentiation in Advanced HCC, Leading to Tumor Regression. Cell Rep (2015) 10(10):1692–707. 10.1016/j.celrep.2015.02.027 PMC4565791 25772357

[29] Wang Y Dong Q Zhang Q Li Z Wang E Qiu X. Overexpression of yes-associated protein contributes to progression and poor prognosis of non-small-cell lung cancer. Cancer Sci (2010) 101(5):1279–85. 10.1111/j.1349-7006.2010.01511.x PMC11158334 20219076

[30] Yoon CI Ahn SG Bae SJ Shin YJ Cha C Park SE. High A20 expression negatively impacts survival in patients with breast cancer. PLoS One (2019) 14(8):e0221721. 10.1371/journal.pone.0221721 31449546 PMC6709902

[31] Hammond ME Hayes DF Dowsett M Allred DC Hagerty KL Badve S. American Society of Clinical Oncology/College Of American Pathologists guideline recommendations for immunohistochemical testing of estrogen and progesterone receptors in breast cancer. J Clin Oncol (2010) 28(16):2784–95. 10.1200/jco.2009.25.6529 PMC2881855 20404251

[32] Wolff AC Hammond MEH Allison KH Harvey BE Mangu PB Bartlett JMS. Human Epidermal Growth Factor Receptor 2 Testing in Breast Cancer: American Society of Clinical Oncology/College of American Pathologists Clinical Practice Guideline Focused Update. J Clin Oncol (2018) 36(20):2105–22. 10.1200/jco.2018.77.8738 29846122

[33] Kanai F Marignani PA Sarbassova D Yagi R Hall RA Donowitz M. TAZ: a novel transcriptional co-activator regulated by interactions with 14-3-3 and PDZ domain proteins. EMBO J (2000) 19(24):6778–91. 10.1093/emboj/19.24.6778 PMC305881 11118213

[34] Sudol M. Yes-associated protein (YAP65) is a proline-rich phosphoprotein that binds to the SH3 domain of the Yes proto-oncogene product. Oncogene (1994) 9(8):2145–52. 8035999

[35] Jeong W Kim SB Sohn BH Park YY Park ES Kim SC. Activation of YAP1 is associated with poor prognosis and response to taxanes in ovarian cancer. Anticancer Res (2014) 34(2):811–7. PMC4082822 24511017

[36] Song M Cheong JH Kim H Noh SH Kim H. Nuclear expression of Yes-associated protein 1 correlates with poor prognosis in intestinal type gastric cancer. Anticancer Res (2012) 32(9):3827–34. 22993325

[37] Wang L Shi S Guo Z Zhang X Han S Yang A. Overexpression of YAP and TAZ is an independent predictor of prognosis in colorectal cancer and related to the proliferation and metastasis of colon cancer cells. PLoS One (2013) 8(6):e65539. 10.1371/journal.pone.0065539 23762387 PMC3677905

[38] Lehn S Tobin NP Sims AH Stal O Jirstrom K Axelson H. Decreased expression of Yes-associated protein is associated with outcome in the luminal A breast cancer subgroup and with an impaired tamoxifen response. BMC Cancer (2014) 14:119. 10.1186/1471-2407-14-119 24559095 PMC3937431

[39] Vlug EJ van de Ven RA Vermeulen JF Bult P van Diest PJ Derksen PW. Nuclear localization of the transcriptional coactivator YAP is associated with invasive lobular breast cancer. Cell Oncol (Dordr) (2013) 36(5):375–84. 10.1007/s13402-013-0143-7 PMC3777165 23949920

[40] Sheen-Chen SM Huang CY Tsai CH Liu YW Wu SC Huang CC. Yes-associated protein is not an independent prognostic marker in breast cancer. Anticancer Res (2012) 32(8):3321–5. 22843909

[41] Cao L Sun PL Yao M Jia M Gao H. Expression of YES-associated protein (YAP) and its clinical significance in breast cancer tissues. Hum Pathol (2017) 68:166–74. 10.1016/j.humpath.2017.08.032 28899737

[42] Lehmann BD Bauer JA Chen X Sanders ME Chakravarthy AB Shyr Y. Identification of human triple-negative breast cancer subtypes and preclinical models for selection of targeted therapies. J Clin Invest (2011) 121(7):2750–67. 10.1172/jci45014 PMC3127435 21633166

[43] Schmid P Adams S Rugo HS Schneeweiss A Barrios CH Iwata H. Atezolizumab and Nab-Paclitaxel in Advanced Triple-Negative Breast Cancer. N Engl J Med (2018) 379(22):2108–21. 10.1056/NEJMoa1809615 30345906

[44] Cortes J Cescon DW Rugo HS Nowecki Z Im SA Yusof MM. Pembrolizumab plus chemotherapy versus placebo plus chemotherapy for previously untreated locally recurrent inoperable or metastatic triple-negative breast cancer (KEYNOTE-355): a randomised, placebo-controlled, double-blind, phase 3 clinical trial. Lancet (2020) 396(10265):1817–28. 10.1016/s0140-6736(20)32531-9 33278935

[45] Jaramillo-Rodriguez Y Cerda-Flores RM Ruiz-Ramos R Lopez-Marquez FC Calderon-Garciduenas AL. YAP expression in normal and neoplastic breast tissue: an immunohistochemical study. Arch Med Res (2014) 45(3):223–8. 10.1016/j.arcmed.2014.01.010 24606817

