# Supplementary material for: High Nuclear Expression of Yes-Associated Protein 1 Correlates With Metastasis in Patients With Breast Cancer
Source: Front Oncol. 2021 Feb 25;11:609743. doi: 10.3389/fonc.2021.609743 (PMC7947190; doi:10.3389/fonc.2021.609743)
Supplement: Supplementary file 1 [file DataSheet_1.docx]

**Supplementary Table 1. Clinical characteristics in relation to nuclear YAP1 expression in the validation cohort**

|  | **YAP1-low, n=428(%)** | **YAP1-high, n=54 (%)** | ***P* value** |
| --- | --- | --- | --- |
| **Age (year, mean±SD)** | 49.39±11.11 | 46.59±10.56 | 0.080 |
| **ER** |  |  | 0.068 |
| Positive | 269 (62.9) | 27 (50.0) |  |
| Negative | 159 (37.1) | 27 (50.0) |  |
| **PR** |  |  | 0.290 |
| Positive | 215 (50.2) | 23 (42.6) |  |
| Negative | 213 (49.8) | 31 (57.4) |  |
| **HER2^a^** |  |  | 0.250 |
| Positive | 74 (17.3) | 6 (11.1) |  |
| Negative | 354 (82.7) | 48 (88.9) |  |
| **HG** |  |  | 0.403 |
| I, II | 286 (66.8) | 33 (61.1) |  |
| III | 142 (33.2) | 21 (38.9) |  |
| **Subtype** |  |  | 0.025 |
| Luminal/HER2(-) | 232 (54.2) | 24 (44.4) |  |
| HER2 (+) | 80 (18.7) | 6 (11.1) |  |
| TNBC | 116 (27.1) | 24 (44.4) |  |
| **Tumor size** |  |  | 0.926 |
| ≤2 cm | 201 (47.0) | 25 (46.3) |  |
| >2 cm | 227 (53.0) | 29 (53.7) |  |
| **Lymph node metastasis** |  |  | 0.121 |
| Negative | 261 (61.0) | 27 (50.0) |  |
| Positive | 167 (39.0) | 27 (50.0) |  |

SD, standard deviation; ER, estrogen receptor; PR, progesterone receptor; HER-2, human epidermal growth factor receptor-2; HG, histologic grade

^a^HER-2 positivity was defined by 3+ on immunohistochemistry or amplification of fluorescence *in situ* hybridization

**Supplementary table 2. Hazard Ratios (HRs) and 95% confidence intervals (CIs) for overall survival (OS)**

|  | **Univariate analysis** | | **Multivariate analysis** | |
| --- | --- | --- | --- | --- |
|  | **HRs (95% CIs)** | ***P* value** | **HRs (95% CIs)** | ***P* value** |
| **Age** | 1.002 (0.958-1.047) | 0.940 |  |  |
| **HR** |  | 0.003 |  | 0.006 |
| Negative | Reference |  | Reference |  |
| Positive | 0.047 (0.006-0.358) |  | 0.059 (0.008-0.447) |  |
| **HER2** |  | 0.280 |  |  |
| Negative | Reference |  |  |  |
| Positive | 0.329 (0.044-2.474) |  |  |  |
| **HG** |  | 0.074 |  |  |
| Ⅰ, Ⅱ | Reference |  |  |  |
| Ⅲ | 1.543 (0.958-2.483) |  |  |  |
| **Tumor size** |  | 0.017 |  | 0.093 |
| ≤2 cm | Reference |  | Reference |  |
| >2 cm | 4.572 (1.319-15.849) |  | 2.915 (0.838-10.145) |  |
| **Lymph node metastasis** |  | 0.053 |  |  |
| Negative | Reference |  |  |  |
| Positive | 2.507 (0.989-6.356) |  |  |  |
| **YAP1 expression** |  | 0.019 |  | 0.343 |
| Low | Reference |  | Reference |  |
| High | 3.035 (1.202-7.665) |  | 1.576 (0.616-4.034) |  |
| **Ki67 (%)** |  | 0.050 |  |  |
| ≤20% | Reference |  |  |  |
| >20% | 3.278 (0.999-10.751) |  |  |  |
| **Lymphovascular invasion** |  | 0.186 |  |  |
| Negative | Reference |  |  |  |
| Positive | 2.022 (0.712-5.742) |  |  |  |
| **TILs** | 0.997 (0.979-1.016) | 0.783 |  |  |

HR, hormone receptor; HER-2, human epidermal growth factor receptor-2; HG, histologic grade; TILs, tumor-infiltrating lymphocytes

**Supplementary Table 3. HRs and 95% CIs for disease-free survival (DFS) in the validation cohort**

|  | **Univariate analysis** | | **Multivariate analysis** | |
| --- | --- | --- | --- | --- |
|  | **HRs (95% CIs)** | ***P* value** | **HRs (95% CIs)** | ***P* value** |
| **Age** | 0.984 (0.959-1.010) | 0.223 |  |  |
| **ER** |  | 0.003 |  | 0.457 |
| Negative | Reference |  | Reference |  |
| Positive | 0.441 (0.256-0.761) |  | 0.750 (0.352-1.598) |  |
| **PR** |  | 0.002 |  | 0.002 |
| Negative | Reference |  | Reference |  |
| Positive | 0.390 (0.214-0.711) |  | 0.374 (0.203-0.689) |  |
| **HER2** |  | 0.084 |  |  |
| Negative | Reference |  |  |  |
| Positive | 1.738 (0.928-3.252) |  |  |  |
| **HG** |  | 0.086 |  |  |
| Ⅰ, Ⅱ | Reference |  |  |  |
| Ⅲ | 1.425 (0.951-2.135) |  |  |  |
| **Tumor size** |  | 0.007 |  | 0.109 |
| ≤2 cm | Reference |  | Reference |  |
| >2 cm | 2.320 (1.255-4.288) |  | 1.671 (0.892-3.132) |  |
| **Lymph node metastasis** |  | <0.001 |  | <0.001 |
| Negative | Reference |  | Reference |  |
| Positive | 2.993 (1.695-5.286) |  | 2.979 (1.669-5.317) |  |
| **YAP1 expression** |  | 0.013 |  | 0.028 |
| Low | Reference |  | Reference |  |
| High | 2.327 (1.197-4.524) |  | 2.112 (1.083-4.119) |  |

ER, estrogen receptor; PR, progesterone receptor; HER-2, human epidermal growth factor receptor-2; HG, histologic grade

**Supplementary table 4. Clinical characteristics in relation to nuclear YAP1 expression in TNBC subtype**

|  | **YAP1-low, n=80 (%)** | **YAP1-high, n=66 (%)** | ***P* value** |
| --- | --- | --- | --- |
| **Age (year, mean±SD)** | 49.33±11.48 | 49.64±10.24 | 0.864 |
| **HG^a^** |  |  | 0.994 |
| Ⅰ, Ⅱ | 21 (26.3) | 17 (25.8) |  |
| Ⅲ | 57 (71.3) | 46 (69.7) |  |
| Missing | 2 (2.5) | 3 (4.5) |  |
| **Tumor size** |  |  | 0.740 |
| ≤2 cm | 30 (37.5) | 23 (34.8) |  |
| >2 cm | 50 (62.5) | 43 (65.2) |  |
| **Lymph node metastasis** |  |  | 0.327 |
| Negative | 57 (71.3) | 42 (63.6) |  |
| Positive | 23 (28.8) | 24 (36.4) |  |
| **Ki67 (%)^a^** |  |  | 0.981 |
| ≤20% | 24 (30.0) | 20 (30.3) |  |
| >20% | 44 (55.0) | 37 (56.1) |  |
| Missing | 12 (15.0) | 9 (13.6) |  |
| **Lymphovascular invasion^a^** |  |  | 0.499 |
| Negative | 60 (75.0) | 48 (72.7) |  |
| Positive | 11 (13.8) | 12 (18.2) |  |
| Missing | 9 (11.3) | 6 (9.1) |  |
| **TILs (%,mean±SD)^a^** | 45.08±32.64 (n=61) | 36.74±34.00 (n=49) | 0.193 |

SD, standard deviation; HG, histologic grade; TILs, tumor-infiltrating lymphocytes

^a^Percentages calculated without missing values

**Supplementary table 5. HRs and 95% CIs for DMFS in the TNBC subtype**

|  | **Univariate analysis** | | **Multivariate analysis** | |
| --- | --- | --- | --- | --- |
|  | **HRs (95% CIs)** | ***P* value** | **HRs (95% CIs)** | ***P* value** |
| **Age** | 0.983 (0.946-1.022) | 0.389 |  |  |
| **HG** |  | 0.586 |  |  |
| Ⅰ, Ⅱ | Reference |  |  |  |
| Ⅲ | 0.889 (0.581-1.359) |  |  |  |
| **Tumor size** |  | 0.114 |  |  |
| ≤2 cm | Reference |  |  |  |
| >2 cm | 2.215 (0.827-5.932) |  |  |  |
| **Lymph node metastasis** |  | 0.013 |  | 0.013 |
| Negative | Reference |  | Reference |  |
| Positive | 2.781 (1.245-6.211) |  | 2.757 (1.233-6.163) |  |
| **YAP1 expression** |  | 0.043 |  | 0.046 |
| Low | Reference |  | Reference |  |
| High | 2.351 (1.027-5.383) |  | 2.329 (1.016-5.339) |  |
| **Ki67 (%)** |  | 0.126 |  |  |
| ≤20% | Reference |  |  |  |
| >20% | 0.475 (0.183-1.232) |  |  |  |
| **Lymphovascular invasion** |  | 0.410 |  |  |
| Negative | Reference |  |  |  |
| Positive | 1.526 (0.559-4.171) |  |  |  |
| **TILs** | 0.988 (0.973-1.004) | 0.130 |  |  |

HG, histologic grade; TILs, tumor-infiltrating lymphocytes
