# Supplementary figures and images for: High Nuclear Expression of Yes-Associated Protein 1 Correlates With Metastasis in Patients With Breast Cancer
Source: Front Oncol. 2021 Feb 25;11:609743. doi: 10.3389/fonc.2021.609743 (PMC7947190; doi:10.3389/fonc.2021.609743)

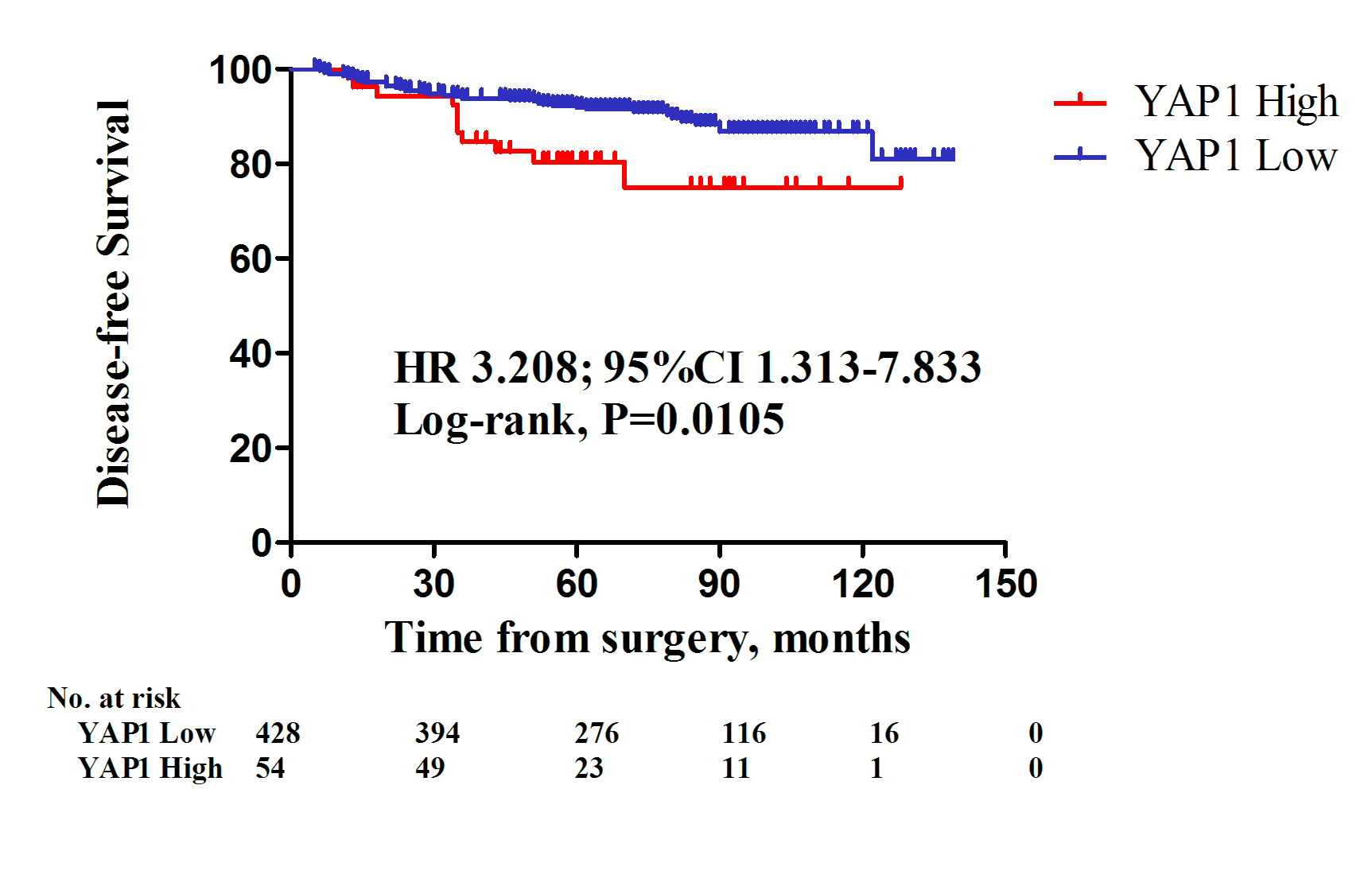

Supplement: Supplementary file 2 [file Image_1.tif]
